# Experimental Research on Dynamic Mechanical Properties of High-Density Foamed Concrete

**DOI:** 10.3390/ma17194781

**Published:** 2024-09-28

**Authors:** Menghui Guo, Yongsheng He, Xudong Zhi

**Affiliations:** 1 Key Lab of Structures Dynamic Behaviour and Control of the Ministry of Education, Harbin Institute of Technology, Harbin 150090, China; guomenghui_edu@sina.com; 2 Key Lab of Smart Prevention and Mitigation of Civil Engineering Disasters of the Ministry of Industry and Information Technology, Harbin Institute of Technology, Harbin 150090, China; 3 Institute of Defense Engineering, AMS, PLA, Luoyang 471023, China; hyshyb2023@163.com

**Keywords:** foamed concrete, dynamic properties, SHPB, strain rate effect, energy absorption characteristics

## Abstract

Foamed concrete is increasingly utilized in protection engineering because it offers a high energy absorption ratio and a relatively low construction cost. To investigate the dynamic properties of foamed concrete, a series of dynamic compression tests are carried out on high-density foamed concrete with densities of 800 kg/m^3^, 1000 kg/m^3^, and 1100 kg/m^3^ under a strain rate range of 59.05 s^−1^~302.17 s^−1^ by using a Φ-100 mm split Hopkinson pressure bar (SHPB) device. The effects of strain rate on the stress–strain relationship, dynamic compressive strength, and dynamic increase factor of foamed concrete are discussed in detail. The results show that the dynamic mechanical characteristics of foamed concrete with different densities exhibit a significant strain rate enhancement effect. Additionally, the energy absorption characteristics of foamed concrete are investigated, demonstrating that it can effectively prevent the transmission of incident energy and that its energy absorption efficiency declines as the strain rate increases. A high-speed camera was also employed to capture the failure process of foamed concrete. The results exhibit that fracture production and development induce the failure of foamed concrete, the failure process of foamed concrete advances as the strain rate increases, and the failure mode becomes increasingly severe.

## 1. Introduction

Porous materials, characterized by their distinctive architecture, exhibit low impedance and high energy absorption ratios, which are instrumental in the effective attenuation of shock waves and in mitigating structural damage under conditions of impact and explosive loading [1]. As a typical porous material, foamed concrete has commendable energy absorption capabilities [2]. Moreover, it is an environmentally friendly construction material due to its low cost and carbon emissions [3,4]. Thus, foamed concrete is regarded as a material of immense potential for structural protection.

Currently, the research on foamed concrete mainly focuses on its physical and static mechanical properties. Numerous experimental results have confirmed the influence of cellular characteristics on the mechanical properties of foamed concrete. The compressive strength increases with decreasing pore diameter and increasing uniformity in distribution [5,6]. Based on the results of uniaxial and triaxial compression experiments, some researchers suggested a set of nonlinear mechanical models to describe foamed concrete under compressive load, accounting for temperature effects and multi-axial mechanical features [7,8,9,10]. In addition, some researchers improved mechanical properties by changing additives or adding fibers into foamed concrete. The results showed that nano-silica fume and fiber reinforcement significantly enhance the compressive and tensile strength of foamed concrete [11,12,13].

In recent years, many scholarly investigations have substantiated the exceptional cushioning and energy absorption characteristics of foamed concrete. Su et al. [14] proposed foamed concrete as a new cushioning material and evaluated it using physical large-scale pendulum impact tests. The results showed that foamed concrete significantly reduces the load acting on the structure and reduces the effect of stress concentration. Guo et al. [15] conducted a series of drop hammer impact tests to analyze the impact buffering effect of foamed concrete as a cushion layer on concrete slabs, and the critical design thickness of the cushion layer was obtained. Zhang et al. [16,17] proposed an analytical model for predicting aircraft gear loads of an engineered material arresting system (EMAS) constructed using foamed concrete. In addition, foamed concrete also exhibits good performance in resisting the destruction of the structure under explosion loads. Wang et al. [18] investigated the confined blast mitigation effectiveness of buried steel boxes with foamed concrete sacrifice panels experimentally and numerically. The results showed that reasonably designed foamed concrete sacrifice panels effectively mitigated confined blasts and reduced structural deformation. Baziar et al. [19] investigated the impact of dynamic loads on underground structures and the performance of foamed concrete as a barrier using centrifugal tests, confirming the effectiveness of foamed concrete in suppressing dynamic loads. The research of Zhou et al. [20] also showed that the foamed concrete buffer layer outside the underground engineering structure can effectively attenuate the explosion loads acting on the structure.

When foamed concrete is used as a protective material, it is mainly faced with high strain rate loads such as impacts and explosions, and more than the traditional quasi-static compression tests are needed to guide the practical application of foamed concrete in protective engineering. Some scholars have conducted dynamic mechanical properties tests of foamed concrete to study its mechanical properties under these circumstances. Jones et al. [21] and Guo et al. [15] conducted a series of low-speed impact experiments to investigate the relationship between the density, water–binder ratio, and energy absorption capacity of foamed concrete, although they did not consider the material’s strain rate effect. Guo et al. [8] investigated the uniaxial compression properties of foamed concrete at the strain rate range of 0.001~118 s^−1^. The results showed that the mechanical properties are more affected by density and temperature but less by strain rate. Wang et al. [22] investigated the failure mode, energy absorption, and strain rate effect of autoclaved aerated concrete (AAC) with a drop hammer impact test, and the study found that the dynamic increase factor (DIF) of AAC increased with the strain rate. In addition, the split Hopkinson pressure bar (SHPB) device test equipment is often used to study the dynamic mechanical properties of materials at high strain rates. Feng et al. [23,24] investigated the strain rate effects of foamed concrete with densities of 300 kg/m^3^, 450 kg/m^3^, and 700 kg/m^3^ by using an SHPB device. The results revealed that the DIF increases linearly with strain rate. In addition, the fractal dimension was proposed to describe the damage characteristics of foamed concrete quantitatively, and the relationship between the fractal dimension and energy absorption was also discussed. Ma et al. [25] conducted SHPB tests on foamed concrete with varying densities, and a conclusion similar to Feng et al. was obtained. However, the dynamic stress–strain curves of foam concrete under high-strain rate loadings were quite different from the results of Feng et al. The SHPB tests of foamed concrete with varying densities (220 kg/m^3^~700 kg/m^3^) conducted in other research also showed the strain rate effect of foamed concrete, but the test results were quite discrete [26,27,28].

The density of foamed concrete is a key factor affecting its compressive strength. Existing studies on the dynamic mechanical properties of foamed concrete mostly focused on low-density (220 kg/m^3^~700 kg/m^3^) foamed concrete, which has a compressive strength usually between 0.4 and 3.0 MPa. Underground protection engineering is usually needed in rock and soil media with high in situ stress. Thus, foamed concrete with higher strength is needed. However, the study on the dynamic mechanical properties of foamed concrete with a density higher than 800 kg/m^3^ is rarely reported. Therefore, in this paper, a series of dynamic compression tests were carried out on foamed concrete with a density of 800 kg/m^3^, 1000 kg/m^3^, and 1100 kg/m^3^ using an SHPB device. The dynamic mechanical and energy absorption properties of foamed concrete were discussed in detail. This research provides helpful guidance for applying foamed concrete in protective engineering structures.

## 2. Materials and Methods

### 2.1. Materials

Foamed concrete was prepared using the pre-foaming method. In this test, specimens with densities of 800 kg/m^3^, 1000 kg/m^3^, and 1100 kg/m^3^ were designed by adjusting the mixture proportions. The mixture proportions of foamed concrete and the physical properties of specific raw materials are shown in Table 1 and Table 2, respectively.

**Figure 1:**
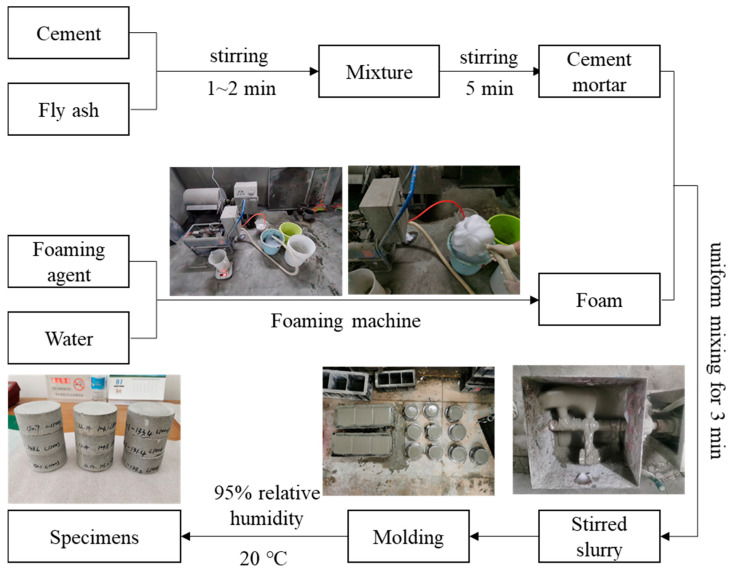
Preparation process of foam concrete.

Figure 1 demonstrates the detailed process of preparing foamed concrete specimens. First, we added the raw cement and fly ash into the mixer according to a specific ratio, shown in Table 1, and evenly stirred for 1~2 min; then, water was added and stirred for 5 min. Meanwhile, the HPL-I foaming agent was used to prepare the required foam which was then quickly added to the mixture, stirring for 3 min. Finally, the stirred slurry was poured into a cylindrical mold with a size of Φ70 mm × 35 mm (for dynamic tests) and a cubic mold with a size of 100 mm × 100 mm × 100 mm (for quasi-static compressive tests), respectively. The specimens attained appropriate strengths after one day of standing, at which point the mold was removed, and the specimens were conventionally cured for 28 days at 20 °C and 95% relative humidity.

### 2.2. Quasi-Static Properties of Foamed Concrete

**Figure 2:**
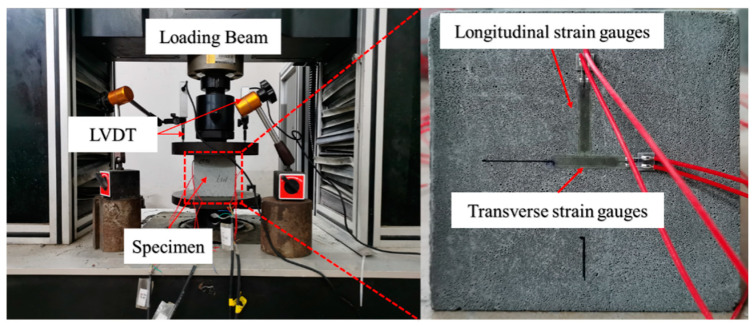
Quasi-static compressive test device.

Quasi-static compressive tests were conducted as per ASTM D1621-16 [29]. Five specimens for each configuration were tested under a loading rate of 2 mm/min with a strain rate of 3.33 × 10^−4^ s^−1^. A pair of transverse and longitudinal strain gauges were pasted on the left and right sides of the specimen, respectively, to gain the compressive strain of the foamed concrete in the elastic stage. At the same time, the axial deformation was monitored by two longitudinal LVDTs arranged after the specimen surface failed. Figure 2 shows the quasi-static compressive test device.

**Figure 3:**
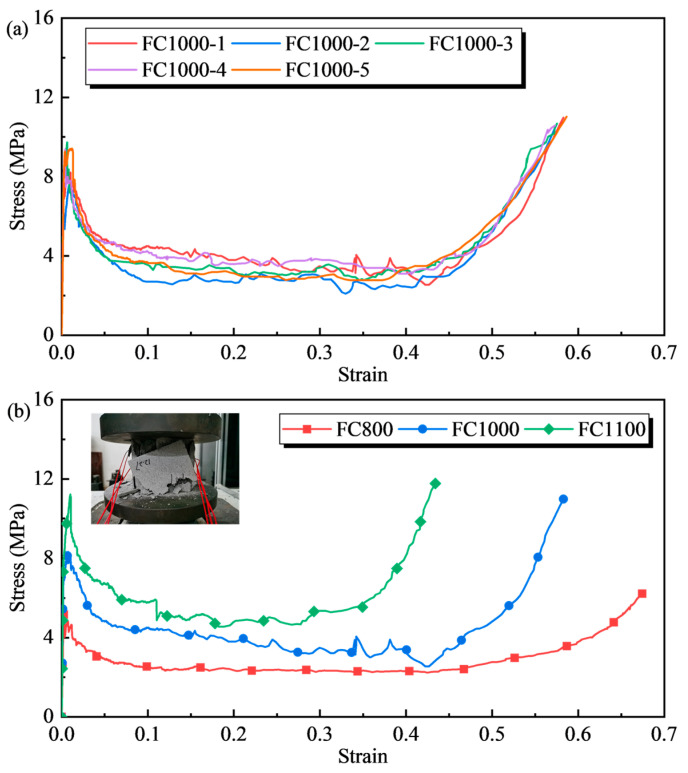
Stress–strain curves of foamed concrete: (**a**) FC1000; (**b**) Typical stress–strain curves of foamed concrete with different densities.

According to the requirements of ASTM D1621-04A, 5 samples were tested for each group of tests to test the uniformity of the samples. Taking the results of FC1000 as an example, the axial compressive stress–strain curve is shown in the Figure 3a, and it can be seen that the test results of each sample have good consistency. Therefore, a sample can be selected for comparison in the subsequent analysis. The typical stress–strain curves of foamed concrete with varying densities under axial compression load are shown in Figure 3b. The stress–strain curve of foamed concrete can be divided into four stages, described below.

Stage 1: Elastic stage. The stress increases linearly with the increase in strain, and the elastic deformation of the cell wall controls the mechanical properties of the foamed concrete. Stage 2: Brittle failure stage. Following the post-peak stress, part of the cell wall reaches the ultimate bearing capacity, and cracks inside the foamed concrete generated through the cavity spread swiftly to the surrounding area. A massive region of the foamed concrete surrounding the cubic specimen peels off, reducing the overall bearing capacity, and the stress–strain curve reveals a sudden drop. Stage 3: Plateau stage. After the stress drops to a specific strength, it does not drop any further, and as the strain continues to increase, it presents as a gentle stress plateau stage. In this stage, the internal cell wall of the foam concrete ruptures and collapses uniformly, and the overall compression of the material occurs. Stage 4: Densification stage. The stress in this stage grows rapidly with an exponential trend.

**Figure 4:**
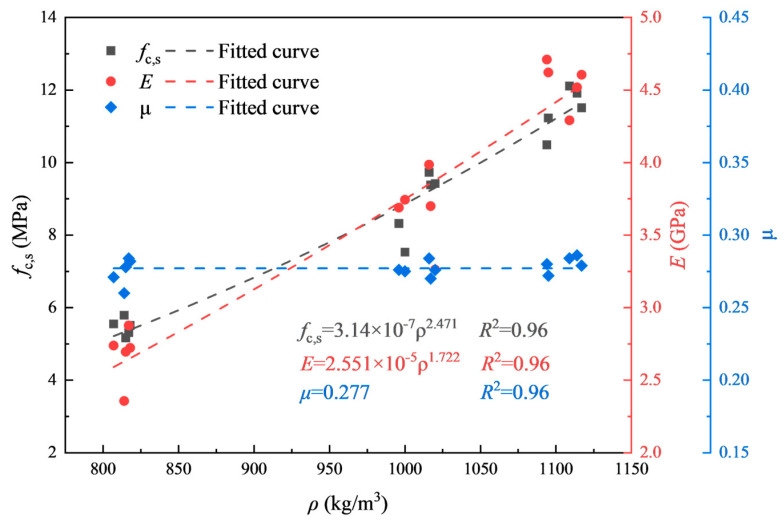
Relationships between mechanical parameters and dry densities.

Table 3 demonstrates the quasi-static physical and mechanical parameters of foamed concretes with different densities. As shown, the standard deviations of each physical and mechanical parameter of foam concrete are slight, indicating that the uniformity of the foamed concrete specimens was acceptable. Figure 4 shows the relationship between the mechanical parameters and the dry density of foamed concrete, which a power function can describe according to Gibson’s research [1]. The axial compressive strength (*f*_c,s_) and elastic modulus (*E*) of foamed concrete increased with dry density (*ρ*), while Poisson’s ratio (*μ*) did not change significantly, which is taken as 0.28 in this study.

### 2.3. Experimental Apparatus and Methods for Dynamic Tests

**Figure 5:**
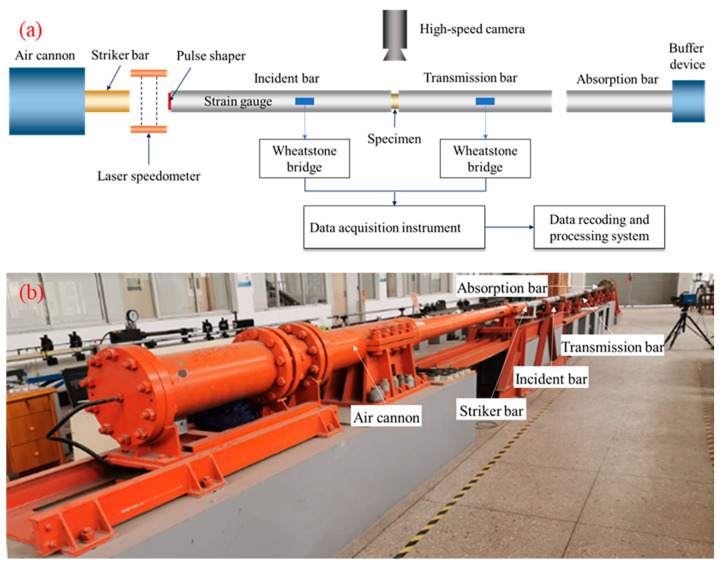
(**a**) Schematic and (**b**) actual set-up of the SHPB system.

Split Hopkinson pressure bar (SHPB) technology is widely used to study the dynamic mechanical properties of materials because it can cover most of the strain rate range caused by explosion and impact loads. This study uses a Φ-100 mm SHPB system to apply uniaxial impact to foamed concrete. As shown in Figure 5, the test system mainly comprises a loading device, compression bar components, and a measuring system. The loading device consists of an aluminum cylindrical striker with a length of 0.6 m and an air cannon. Firstly, the air cannon accelerates the striker through a sudden release of high-pressure gas and then impacts the incident bar to form an incident shock wave to complete dynamic loading. The maximum emission pressure is about 8 MPa, and the impact speed was adjusted by controlling different emission pressures. The compression bar components had incident, transmitted, and absorption bars with lengths of 4.0 m, 3.0 m, and 1.8 m, respectively, made of aluminum alloy with a mass density of 2700 kg/m^3^ and elastic modulus of 70 GPa. Both bars were supported on the specific ball bearing to reduce friction during dynamic tests. The end of the absorption bar is a specially designed damping buffer to eliminate the residual kinetic energy. The measuring system includes a laser speedometer, strain gauges, a dynamic acquisition instrument, and a high-speed camera. The HG202A laser speedometer is placed at the incident bar’s impact end to test the striker’s impact speed. In the SHPB test, the transmitted signal will be fragile because the acoustic impedance of the soft material is much smaller than that of the compression bars [30]. Under the premise of a reasonable signal-to-noise ratio, conventional strain gauges are not sensitive enough to measure the transmitted signal. Semiconductor strain gauges with a high sensitivity of 110 ± 5% and a resistance value of 120 ± 8% Ω were used to test the transmitted signal during the test. The strain gauge type is SB3.8-120-P-2, produced by Zhonghang Electronic Measuring Instruments Co., Ltd. (Hanzhong, China). The strain gauge attachment position on the incident bar and transmitted bar is 1.5 m and 0.5 m away from the section of the incident bar/specimen and specimen/transmitted bar, respectively. The acquisition instrument type is DH5960G, an ultra-high-speed dynamic acquisition instrument, and the sampling frequency is 1 MHz. The data recording and processing adopted DHDAS4.1.3 dynamic signal acquisition and analysis software.

**Figure 6:**
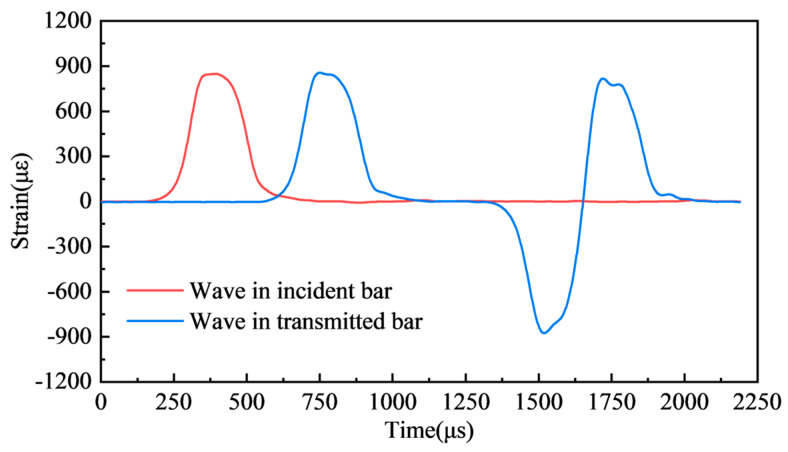
Wave history of a pre-test without a specimen.

According to Chen and Song [31], the wavelength (λ) is about twice the striker length when the striker is a cylinder. With the increased bar radius (r), the transverse dispersion effect in the bar will gradually intensify, where r/λ ≤ 0.1, and the dispersion effect of the wave in the pressure bars can be ignored [32]. In this research, the r/λ ratio equals 0.042 (where the radius equals 50 mm, and the wavelength equals 1200 mm). In addition, a pre-test without a specimen was carried out to check the alignment of the bars before the formal test. The emission pressure is 0.5 MPa, and the strain signals are shown in Figure 6. The shape of the transmitted wave is very close to that of the incident wave, confirming the reliability of the test system.

**Figure 7:**
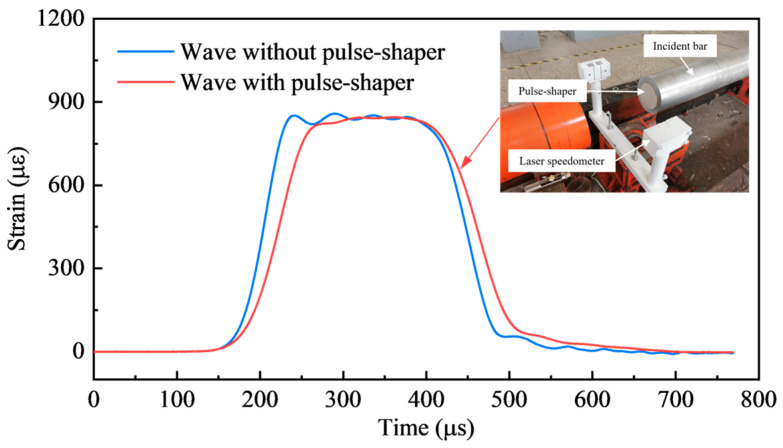
The installation diagram and shaping effect of the cardboard pulse-shaper.

Meanwhile, cardboard was used as a shaper to eliminate the high-frequency stress caused by the collision between the striker bar and the incident bar during the test [33]. The installation diagram of the shaper and the shaping effect of the incident wave are shown in Figure 7. It can be seen that not only is the high-frequency component’s filtering realized, but also the rising time of the incident pulse is effectively improved, which is conducive to the realization of the dynamic stress equilibrium of the specimen.

**Figure 8:**
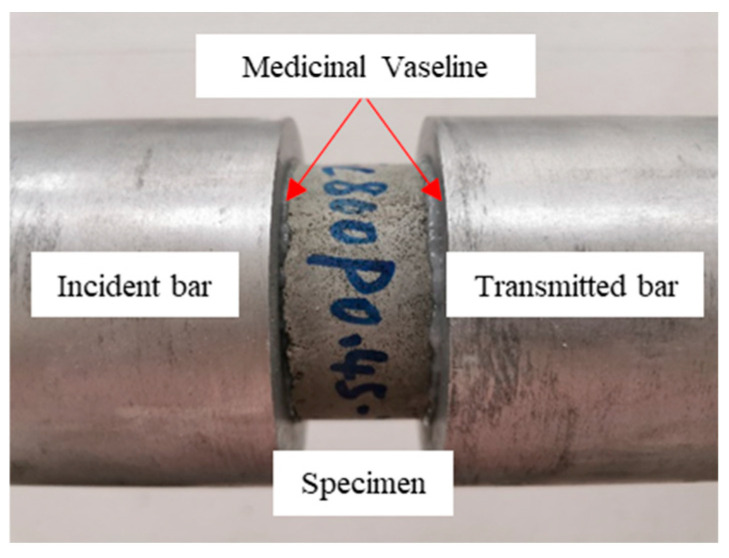
Medicinal Vaseline on the surfaces of specimens and bars.

In addition, a thin layer of grease (where medicinal Vaseline was used) was applied on the contact surfaces between the specimens and the pressure bars during the test to eliminate the influence of the frictional effect, as shown in Figure 8.

### 2.4. Data Processing

#### 2.4.1. Three-Wave Analysis Method

**Figure 9:**
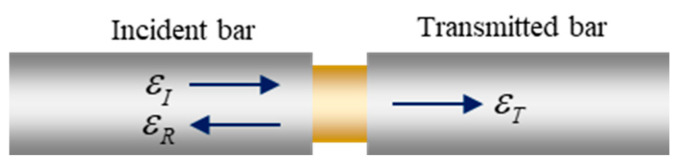
Principle of pulse wave propagation in the SHPB test.

According to the one-dimensional wave propagation theory, the diagram of pulse wave propagation in the SHPB test is shown in Figure 9, where $\varepsilon_{I}$, $\varepsilon_{R}$, and $\varepsilon_{T}$ are the incident, reflected, and transmitted strain wave signals, respectively. The three-wave method is one of the most commonly applied methods for processing test data [31]. Equations (1)–(3) present the calculation formulas of the stress, strain, and strain rate.

(1) $$\sigma =\frac{A_{B}E_{B}}{2A_{S}}\left(\varepsilon_{I}+\varepsilon_{R}+\varepsilon_{T}\right)$$

(2) $$\dot{\varepsilon }=\frac{C_{B}}{L_{S}}\left(\varepsilon_{I}-\varepsilon_{R}-\varepsilon_{T}\right)$$

(3) $$\varepsilon =\frac{C_{B}}{L_{S}}\int_{0}^{t}\left(\varepsilon_{I}-\varepsilon_{R}-\varepsilon_{T}\right)dt$$

where σ, $\dot{\varepsilon }$, and ε are the dynamic compressive stress, strain rate, and strain, respectively $A_{B}$, $E_{B}$, and $C_{B}$ are the cross-sectional area, elastic modulus, and P-wave velocity of the bars, respectively. $A_{S}$ and $L_{S}$ are the cross-sectional area and length of the specimens, respectively.

#### 2.4.2. Determination of the Energy Parameters

The energy absorbed by the foamed concrete in the axial dynamic compression test mainly includes the fracture energy (produced by new cracks), heat, acoustic energy, and the kinetic energy of the crushed fragments. However, the latter two portions were proven to be inconsequential due to their lower values [34]. The incident, reflected, and transmitted energy of the stress waves are determined by Equations (4)–(6). Furthermore, assuming that the energy loss of the pressure bar/surface is neglectable, the energy absorbed by specimens can be estimated as Equation (7).

(4) $$W_{I}=C_{0}A_{0}E_{0}\int_{0}^{\tau }\varepsilon_{I}^{2}$$

(5) $$W_{R}=C_{B}A_{B}E_{B}\int_{0}^{\tau }\varepsilon_{R}^{2}$$

(6) $$W_{T}=C_{B}A_{B}E_{B}\int_{0}^{\tau }\varepsilon_{T}^{2}$$

(7) $$W_{A}=W_{I}-W_{R}-W_{T}$$

### 2.5. Experimental Scheme

A total of 30 sets of dynamic impact mechanical properties testing for foamed concrete with three designed densities of 800 kg/m^3^, 1000 kg/m^3^, and 1100 kg/m^3^ were tested in this experiment. Each set of tests evaluated three specimens to guarantee the correctness of the results. Table 4 shows the SHPB dynamic impact test conditions.

## 3. Results and Discussion

### 3.1. Validity and Strain Rate Determination of the Test

**Figure 10:**
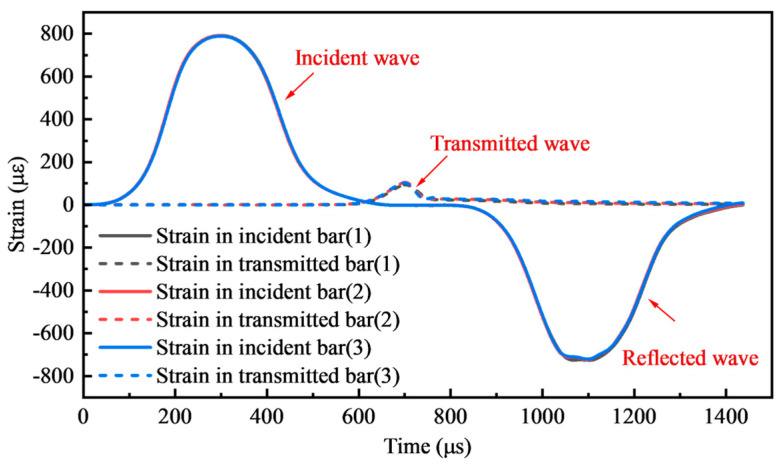
Typical strain wave signals.

Figure 10 shows the typical strain wave signals of the three groups of parallel samples in the impact test of foamed concrete with 5 MPa under 0.15 MPa pressure loading. The semiconductor strain gauges directly detected the bar’s strain signals, which are smooth and low-noise. The strain signal was gathered despite the modest transmitted wave signal (peak strain is nearly 1/8 of the incident wave peak). The strain signals of the three sets of specimens match at the same loading pressure, confirming the stability of the test device.

**Figure 11:**
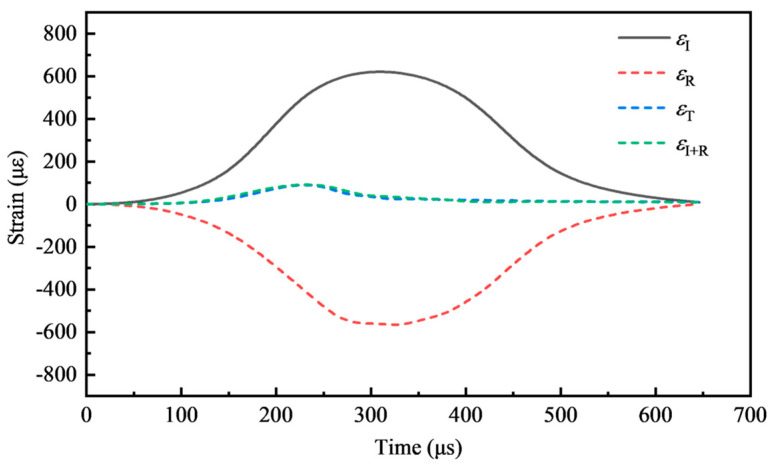
Typical stress equilibrium.

Dynamic stress equilibrium is another fundamental assumption for ensuring the efficacy of SHPB test results. It requires that the stress at both ends of the specimen be equal, which may be validated by the time history curve of the waveform [33]. The dynamic stress equilibrium of all SHPB tests was confirmed by the excellent agreement between $\varepsilon_{T}$ and $\varepsilon_{I}+\varepsilon_{R}$, as shown in Figure 11.

**Figure 12:**
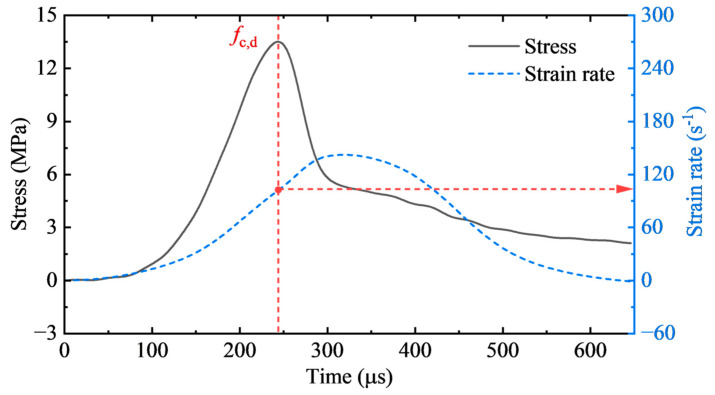
Strain rate determination.

There are several methods to determine the representative strain rate in the SHPB test. As reported in [35,36], the average strain rate was employed as the representative strain rate. In addition, Li et al. [37] used the strain rate at the peak stress point as the representative strain rate. Moreover, a nearly constant strain rate has also been used [32]. The third method is the most exact but the hardest to obtain, whereas employing the average strain rate usually leads to significant errors. In this study, the strain rate corresponding to the peak stress was utilized as the representative strain rate in the test, as shown in Figure 12.

### 3.2. Dynamic Stress–Strain Curves

**Figure 13:**
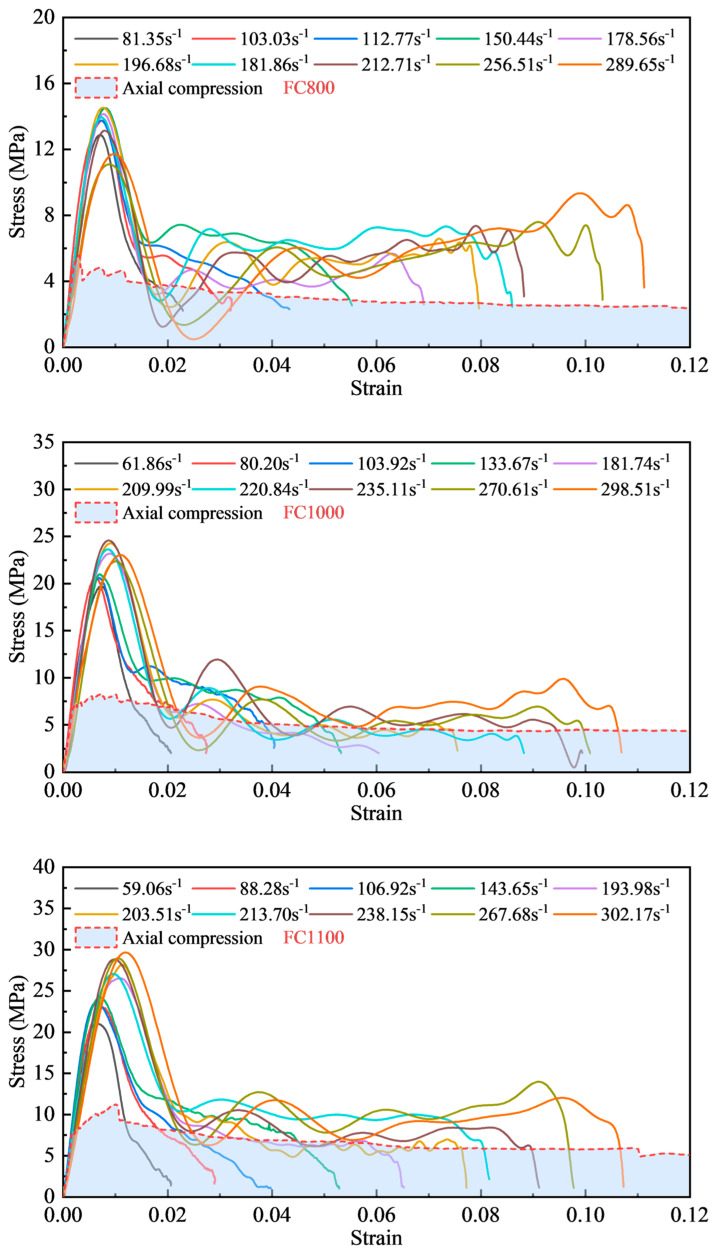
Stress–strain curves of specimens with different densities.

Figure 13 shows the stress–strain relationships for foamed concrete with different densities at a strain rate of 60 s^−1^ to 300 s^−1^, and the peak stress of the stress–strain curve was defined as the dynamic compressive strength ($f_{c,d}$) of the specimens. The dynamic compression strength was more significant than the static axial compression strength, indicating a considerable strain rate effect. Achieving a higher loading strain rate during the SHPB test required a higher striker bar velocity, which resulted in a significant increase in incident energy and, thus, a greater compressive deformation of the foamed concrete. Furthermore, the ultimate strain of the specimens gradually increased with the increase in strain rate.

**Figure 14:**
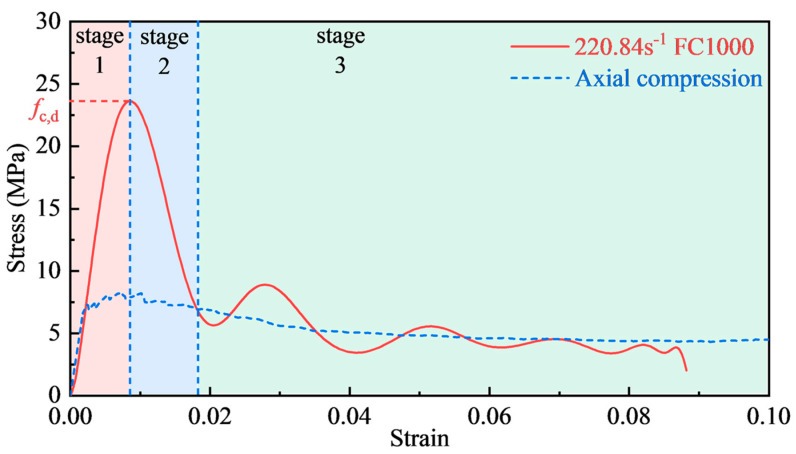
Typical three stages of the stress–strain curve.

Under high strain rate conditions, the higher incident energy allowed the foamed concrete specimens to undergo a more substantial deformation. Typical dynamic stress–strain curves of foamed concrete may be roughly separated into three stages, as shown in Figure 14. The first stage is the load-rising stage. The stress–strain curve grew linearly at the beginning, the impact load was borne by the pore skeleton of foamed concrete, and the material underwent an elastic deformation. Then, the stress quickly exceeded the static axial compression strength with the strain rate effect. When the stress grew to 80% of the peak stress, the specimen began to show a plastic deformation and part of the cell walls bent or even ruptured, but the specimen remained intact until reaching the peak stress. The second stage involved a sudden drop in load. The stress reduced as the strain increased after the peak stress. At this stage, the cellular walls within the foamed concrete were shattered in various ways. Then, the fracture surfaces were subsequently connected, resulting in a significant number of longitudinal cracks, which caused a sharp decline in the bearing capacity. The third stage of dynamic stress–strain occurred when the stress descended to the point where it intersected the quasi-static axial stress–strain curve. The longitudinal cracks were fully developed, and the pore wall collapsed uniformly layer by layer, producing a fluctuating linear stress–strain curve.

### 3.3. Dynamic Properties of Foamed Concrete

**Figure 15:**
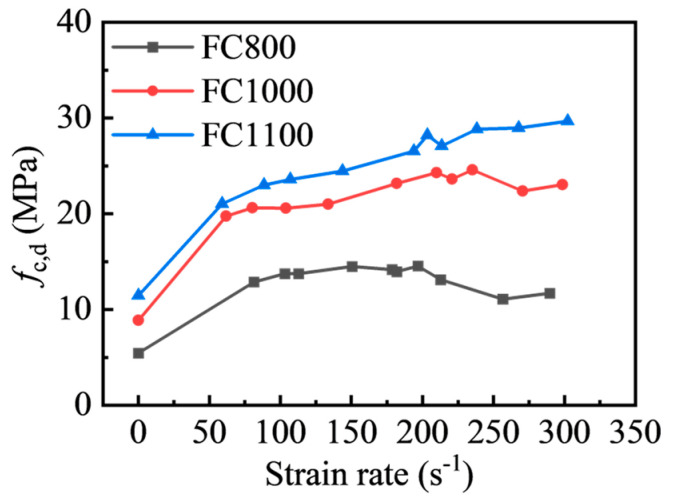
Relationship between the strain rate and peak stress.

Table 4 shows the foamed concrete’s dynamic compressive strength and strain rate under each condition. Figure 15 illustrates the axial compression strength versus strain rate for different densities of foamed concrete. As shown, the dynamic compressive strength increased dramatically compared to quasi-static axial compressive strength, i.e., at strain rates of roughly 60 s^−1^, foamed concrete with densities of 800 kg/m^3^, 1000 kg/m^3^, and 1100 kg/m^3^ was enhanced by 135%, 122%, and 83.7%, respectively. However, when the strain rate continued to increase, the degrees of change in the dynamic compression strength of foamed concrete decreased significantly in the strain rate range of 60 s^−1^~300 s^−1^. Compared to the dynamic compression strength at 60 s^−1^, the strength of foamed concrete with densities of 800 kg/m^3^, 1000 kg/m^3^, and 1100 kg/m^3^ only increased by 11.33%, 19.64%, and 29.11%, and the maximum dynamic compression strength occurs at strain rates of 196.68 s^−1^, 235.11 s^−1^, and 302.17 s^−1^, respectively. The peak stress of FC800 and FC1000 foamed concrete decreases to some extent when the strain rate exceeds 200 s^−1^ and 235 s^−1^, which could be due to the brittle failure of low-strength foamed concrete in advance under the high-speed impact, and the spalling of sample blocks results in low-pressure data in the test.

**Figure 16:**
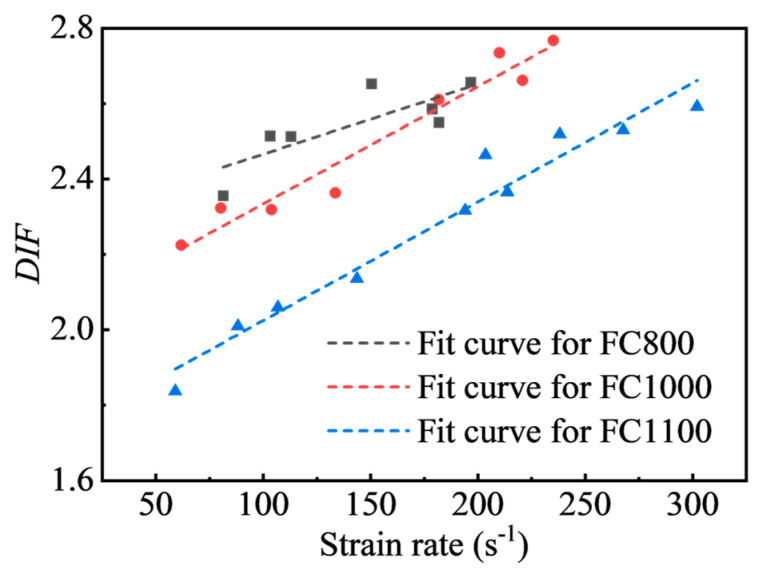
Relationship between *DIF* and strain rate.

The strain rate effect on the dynamic compressive strength of materials under impact loading is commonly quantified by the dynamic increase factor (*DIF*) derived by Equation (8):

(8) $$DIF=f_{c,d}/f_{c,s}$$

where $f_{c,d}$ and $f_{c,s}$ are the dynamic compressive strength and static compressive strength, respectively. Figure 16 demonstrates the *DIF* versus strain rate of foamed concrete with various densities. After discarding data that might be inaccurate, *DIF* increases linearly with increasing strain rate, showing an obvious strain rate enhancement effect, which is consistent with the results in the literature [24]. The predicted equations for *DIF* values versus strain rate for foamed concrete may be derived by fitting the data in Figure 16, as shown in Equations (9)–(11).

(9) $$\mathrm{FC}800\text{:}\;DIF=1.859\times 10^{-3}\times \dot{\varepsilon }+2.280\;(R^{2}=58.32)\;81.35\leq \dot{\varepsilon }\leq 181.86$$

(10) $$\mathrm{FC}1000\text{:}\;DIF=3.113\times 10^{-3}\times \dot{\varepsilon }+2.023\;(R^{2}=94.40)\;61.86\leq \dot{\varepsilon }\leq 235.11$$

(11) $$\mathrm{FC}1100\text{:}\;DIF=3.148\times 10^{-3}\times \dot{\varepsilon }+1.710\;(R^{2}=94.98)\;59.06\leq \dot{\varepsilon }\leq 302.17$$

### 3.4. Energy Absorption Characteristic

The energy absorption characteristics are important mechanical property indices to evaluate the protection capability of porous materials. The incident energy (*W_I_*), reflected energy (*W_R_*), transmitted energy (*W_T_*), and absorbed energy (*W_A_*) of the specimens were calculated for each test according to Equations (4)–(7) in Section 2.4, as shown in Table 4.

**Figure 17:**
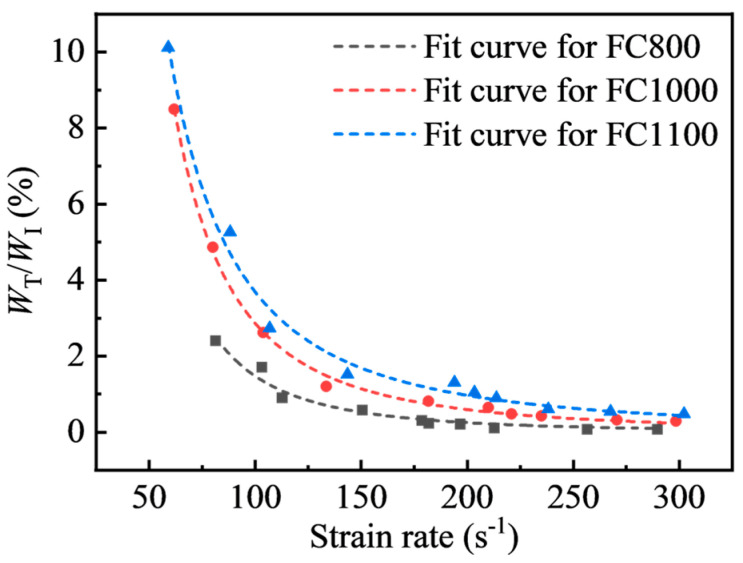
Relationship between the energy transmission coefficient and strain rate.

The energy transmission coefficient is defined as the ratio of the transmitted wave energy to the incident wave energy. Figure 17 shows the energy transmission coefficient of each specimen at different strain rates. It can be seen that the transmitted energy was small during the test, and the maximum was only 10.12% of the incident energy. In addition, the energy transmission coefficient decayed with the increase in the strain rate as a power function. Moreover, it decreased significantly with the decreased density of foamed concrete. This indicates that low-density foamed concrete with larger porosity could reduce energy transmission more effectively.

**Figure 18:**
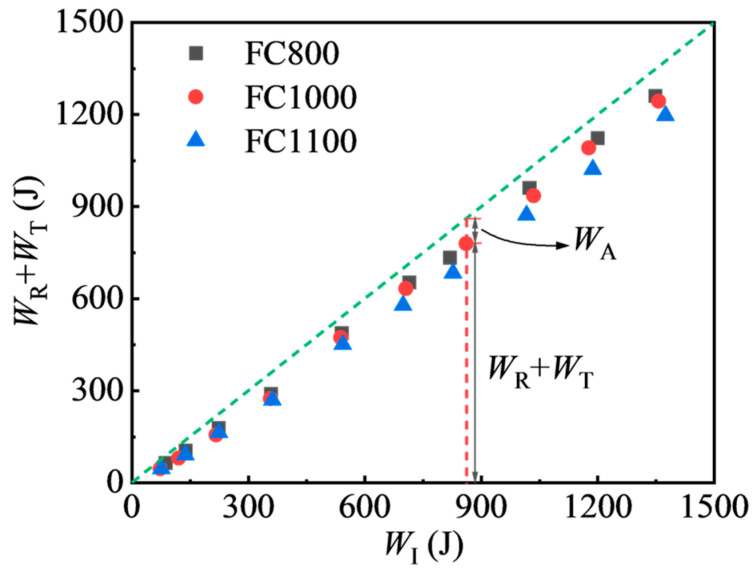
Variations in unused energy of foamed concrete.

The value of reflected energy used to be large in Table 4, which means most of the incident energy in the SHPB test is reflected. Since reflected and transmitted energy is not involved in the damage of specimens, *W_R_* + *W_T_* is also referred to as unused energy. Figure 18 demonstrates that the unused energy increased with the incident energy. All data points were below the diagonal line, indicating that all specimens absorbed impact energy effectively during the impact compression process. The distance between the data point and the diagonal line presents the absorbed energy. As shown, the values of *W_A_* steadily increased as *W_I_* increased. Furthermore, the energy absorbed by foamed concrete increases with density when the incident energy remains constant.

**Figure 19:**
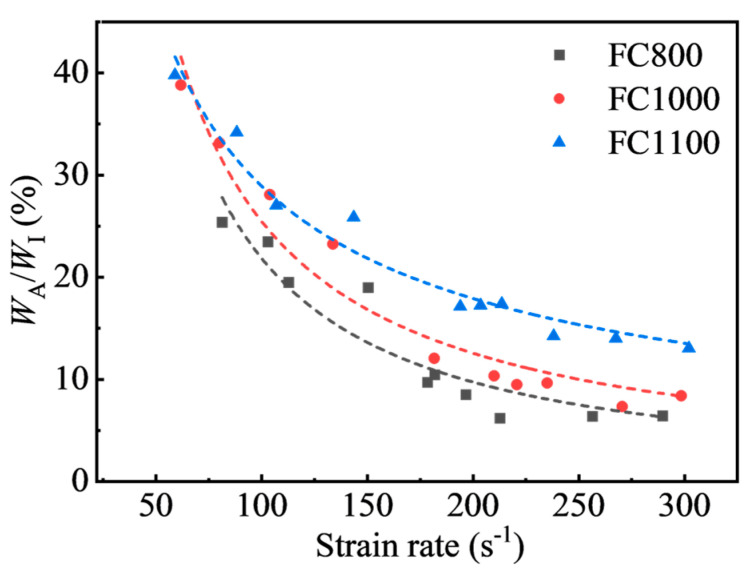
The energy absorption efficiency of foamed concrete.

To quantitatively evaluate the energy absorption capacity of foamed concrete, the energy absorption efficiency was defined as the ratio of *W_A_*/*W_I_*. Although the energy absorbed by the specimen increased with the incident energy, the energy absorption efficiency presents a decreasing tendency, as shown in Figure 19. The incident energy varies from 73.30 J to 1374.99 J in this study. The maximum energy absorption efficiencies for FC800, FC1000, and FC1100 are 25.37%, 38.79%, and 39.75%, respectively. This shows that the density of foamed concrete has a great impact on the energy absorption efficiency. When subjected to high-speed impact loading, foamed concrete lacks sufficient time for deformation, and energy is primarily absorbed through strength failure. High-density foamed concrete demonstrates superior energy absorption efficiency under impact loading due to its higher strength. Due to the limitation of the test equipment, as the strain rate increases, there is a significant increase in incident energy, while the foam concrete absorbs only a limited amount of energy. The majority of incident energy is reflected and transmitted, leading to a decreasing trend in energy absorption efficiency as the strain rate increases.

### 3.5. Failure Process and Patterns

**Figure 20:**
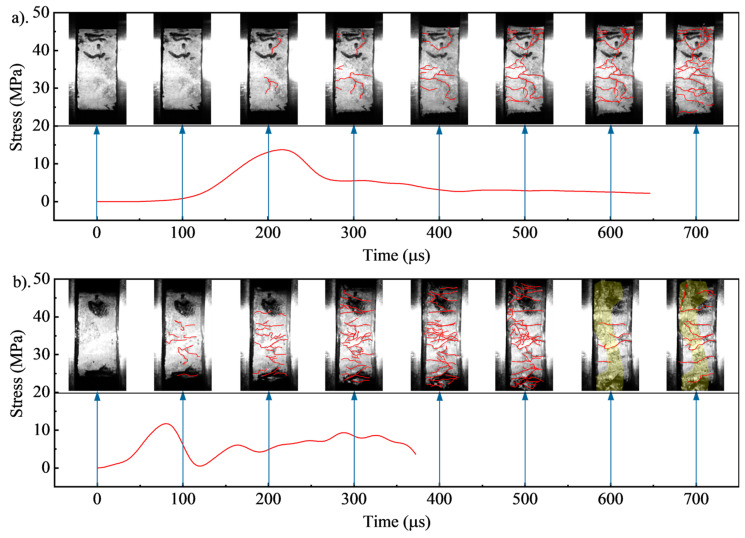
Failure process of foamed concrete: (**a**) FC800 at 103.03 s^−1^, (**b**) FC800 at 289.65 s^−1^.

A high-speed camera recorded the failure process of foamed concrete at 20,000 frames per second. Figure 20 shows the failure process of FC800 with a strain rate of 103.03 s^−1^ and 289.65 s^−1^. For a strain rate of 103.03 s^−1^, as shown in Figure 20a, the specimen was uniformly compressed during 0~100 μs, and no cracks occurred due to low stress. Vaseline was extruded at the end of the specimens, and the pressure bars were in complete contact with the specimens. The stress reached peak stress around 200 μs, and then micro-cracks occurred initially at the defect position in the middle of the specimen. The stress increased rapidly, while little cracks developed during 100~200 μs. In this stage, the absorbed energy was mainly caused by elastic deformation. At 200~300 μs, the stress decreased from the peak value to the platform’s stress section, and fracture development was minimal. At 300 μs, a partial surface crack occurred at the contact surface between the specimen and the incident bar. Then, it gradually connected with the crack in the middle until it penetrated the whole specimen. The fissures on the specimen’s surface expanded fast in stage 3 at 300~700 μs, and at 700 μs, the specimen split into multiple small pieces, with some debris expelled. Figure 20b shows the damage process of the specimen at 289.65 s^−1^. The effective duration of the stress action declined with the increasing strain rate, and the peak stress moment occurred earlier than 103.03 s^−1^. At 100 μs, the specimen exceeded the peak stress with several longitudinal cracks in the middle of the specimen. Then, at 200 μs, the longitudinal cracks penetrated the entire specimen, which was broken into several small fragments. After 200 μs, the crushed foamed concrete is not entirely separated and continues to be uniformly compressed under the impact load, resulting in a gradually rising stress. Local foamed concrete was compacted at this stage, and the fragments were broken into smaller pieces. However, when the time reached 600 μs, crack development on the specimen’s surface was no longer visible. The original foamed concrete fragments were crushed entirely into uniform granular shapes in the middle region of the specimen, forming a wholly crushed area, depicted by the yellow-shaded part in Figure 20b.

**Figure 21:**
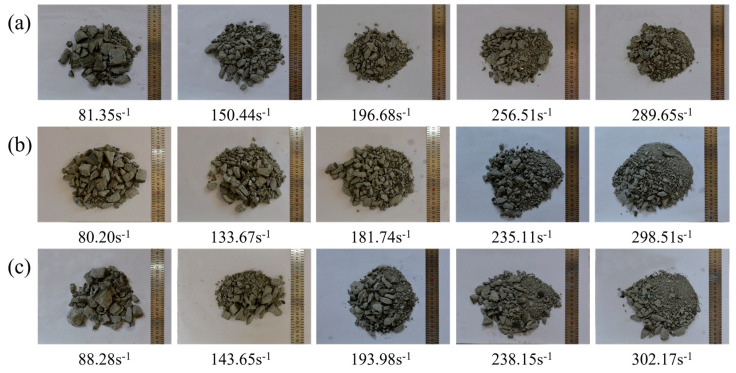
Failure modes of foamed concrete: (**a**) FC800; (**b**) FC1000; (**c**) FC1100.

Figure 21 shows the failure patterns of foamed concrete with various strengths. As observed, the rising strain rate significantly affected the failure patterns of the specimens, which the strain rate hardening effect could explain. The dynamic compressive strength and absorbed energy increased with the rise in the strain rate. The generation and expansion of cracks in the specimen indicated the damage of foamed concrete generated by impact loading. Furthermore, the foamed concrete breaks into smaller and smaller fragments as the impact strain rate increases. Foamed concrete was partially broken into powder as the strain rate exceeded 230 s^−1^. With similar strain rate conditions, the damage modes of foamed concrete with different densities in this research are very similar.

## 4. Conclusions

In this paper, a series of dynamic compression tests are carried out on foamed concrete with densities of 800 kg/m^3^, 1000 kg/m^3^, and 1100 kg/m^3^ under a strain rate range of 59.05 s^−1^~302.17 s^−1^ by using a Φ-100 mm SHPB system. The dynamic properties, energy characteristics, failure process, and modes of foamed concrete are experimentally investigated. The main conclusions are summarized as follows:

(1) The dynamic stress–strain curves of foamed concrete include elastic deformation, decreasing stress, and residual stress stages.
(2) The dynamic compressive strength of foam concrete shows a substantial strain rate correlation, which is much higher than the static compressive strength in the test strain rate range of 59.05 s^−1^~302.17 s^−1^. Within the tested strain rate range, the dynamic increase factor of foamed concrete can be expressed by a linear function of the strain rate.
(3) Foamed concrete can effectively prevent energy transmission with a maximum energy transmission coefficient of 10.12%, and the transmission coefficient shows exponential decay as the strain rate rises. Meanwhile, the transmission coefficient declines with increasing dry density. Moreover, the energy absorption efficiency of foamed concrete reduces steadily with increasing strain rate, and the maximum energy absorption rate of foamed concrete is 25.37%, 38.79%, and 39.75% for FC800, FC1000, and FC1100, respectively.
(4) The strain rate considerably affects the failure process and failure modes of foamed concrete.

## Figures and Tables

**Table 1:** The mixture proportions of foamed concrete with different axial compression strengths.

Group ^1^	Component (kg/m^3^)				
	Cement	Fly Ash	Water-Reducing Agent	Foaming Agent	Water
FC800	436.0	218.0	2.6	0.389	179.7
FC1000	560.0	240.0	4.5	0.360	201.8
FC1100	600.0	300.0	3.6	0.341	245.1

^1^ FC800, FC1000, and FC1100 indicate that the design density of foamed concrete is 800 kg/m^3^, 1000 kg/m^3^, and 1100 kg/m^3^, respectively. W/C ratio for FC800, FC1000, and FC1100 is 0.41, 0.36, and 0.40, respectively.

**Table 2:** Physical properties of specific raw materials.

Materials	Physical Properties	Manufacturer
Cement	P.O 42.5R (GB 175-2007) Density = 3100 kg/m^3^	Guangzhou Zhujiang Cement Co., Ltd., Guangzhou, China
Fly ash	Class II (GB/T 50146-2014) Density = 2.28 kg/m^3^ Fineness = 1.5%	Guangzhou Pearl River Power Plant, Guangzhou, China
Foaming agent	HTL-I Expansion ratio = 26 Defoaming time = 6.5 h	Huatai Building Materials Co., Ltd., Henan, China
Water-reducing agent	QL-PC2 Concentration = 16% Water-reducing rate = 25%	Qiangli Construction Materials Co., Ltd., Guangzhou, China

**Table 3:** Physical and mechanical parameters of foamed concrete.

Group	*ρ* /kg·m^3^	SD-*ρ*	*f*_c,s_ /MPa	SD-*f*_c,s_	*E* /GPa	SD-*E*	*μ*	SD-*μ*
FC800	814	4.32	5.47	0.24	2.678	0.193	0.28	0.009
FC1000	1009	10.96	8.88	0.92	3.676	0.261	0.28	0.005
FC1100	1105	10.71	11.45	0.64	4.549	0.159	0.28	0.005

**Table 4:** Details of the dynamic compression tests in SHPB.

Group	*ρ* (kg/m^3^)	*P* (MPa)	*v* (m/s)	$\dot{\varepsilon }\;$(s^−1^)	*f*_c,d_ (MPa)	DIF	*W_I_* (J)	*W_R_* (J)	*W_T_* (J)	*W_A_* (J)
FC800-0.08	775.33	0.08	4.27	81.35	12.886	2.356	86.58	62.53	2.08	21.97
FC800-0.10	771.44	0.1	5.34	103.03	13.752	2.514	137.44	102.79	2.36	32.29
FC800-0.15	778.79	0.15	6.99	112.77	13.747	2.513	223.11	177.55	2.04	43.52
FC800-0.20	766.21	0.2	8.44	150.44	14.511	2.653	357.68	287.71	2.10	67.87
FC800-0.25	777.46	0.25	9.61	178.56	14.149	2.587	540.53	486.33	1.68	52.51
FC800-0.30	776.58	0.3	11.54	196.68	14.533	2.657	713.77	651.56	1.57	60.65
FC800-0.35	781.31	0.35	11.89	181.86	13.950	2.550	819.36	731.91	1.92	85.53
FC800-0.40	788.79	0.4	14.08	212.71	13.139	2.402	1024.13	959.46	1.13	63.54
FC800-0.45	765.24	0.45	15.00	256.51	11.096	2.029	1200.09	1122.51	0.97	76.61
FC800-0.50	772.11	0.5	16.63	289.65	11.719	2.142	1347.35	1259.58	1.07	86.69
FC1000-0.08	1037.89	0.08	4.02	61.86	19.751	2.224	73.30	38.64	6.22	28.44
FC1000-0.10	984.75	0.1	5.00	80.2	20.625	2.323	120.18	74.51	5.84	39.82
FC1000-0.15	983.57	0.15	7.07	103.92	20.593	2.319	217.48	150.73	5.70	61.05
FC1000-0.20	975.15	0.2	8.49	133.67	20.981	2.363	357.06	269.81	4.31	82.94
FC1000-0.25	1038.64	0.25	9.64	181.74	23.179	2.610	538.21	468.92	4.40	64.90
FC1000-0.30	1028.55	0.3	10.63	209.99	24.279	2.734	705.50	628.03	4.57	72.90
FC1000-0.35	1031.96	0.35	11.89	220.84	23.630	2.661	860.95	775.45	4.08	81.41
FC1000-0.40	1038.64	0.4	13.83	235.11	24.577	2.768	1034.55	930.62	4.39	99.55
FC1000-0.45	982.14	0.45	15.26	270.61	22.357	2.518	1176.70	1086.83	3.77	86.09
FC1000-0.50	989.3	0.5	16.64	298.51	23.038	2.594	1356.79	1239.42	3.88	113.49
FC1100-0.08	1097.7	0.08	4.17	59.06	21.034	1.837	75.68	37.93	7.66	30.09
FC1100-0.10	1081.95	0.1	5.32	88.28	23.004	2.009	138.23	83.71	7.27	47.25
FC1100-0.15	1095.47	0.15	6.71	106.92	23.579	2.059	224.26	157.56	6.13	60.57
FC1100-0.20	1101.19	0.2	8.40	143.65	24.449	2.135	362.52	263.35	5.50	93.66
FC1100-0.25	1112.37	0.25	9.60	193.98	26.527	2.317	543.60	443.37	7.09	93.14
FC1100-0.30	1099.4	0.3	10.46	203.51	28.201	2.463	698.26	570.59	7.33	120.34
FC1100-0.35	1078.39	0.35	11.94	213.7	27.076	2.365	827.17	675.98	7.41	143.79
FC1100-0.40	1097.54	0.4	14.06	238.15	28.834	2.518	1016.76	865.85	6.20	144.71
FC1100-0.45	1075.87	0.45	15.21	267.68	28.958	2.529	1186.95	1014.19	6.43	166.33
FC1100-0.50	1105.74	0.5	16.58	302.17	29.671	2.591	1374.99	1189.18	6.54	179.27

## Data Availability

The data presented in this study are available on request from the corresponding author. The data are not publicly available due to privacy reasons.
